# The seroprevalence and kinetics of IgM and IgG in the progression of COVID-19

**DOI:** 10.1186/s12865-021-00404-0

**Published:** 2021-02-17

**Authors:** Xuzhen Qin, Jun Shen, Erhei Dai, Haolong Li, Guodong Tang, Lixia Zhang, Xin Hou, Minya Lu, Xian Wu, Simeng Duan, Jingjia Zhang, Man-Fung Tsoi, Ping Jiang, Yongzhe Li

**Affiliations:** 1 Department of Laboratory Medicine, Peking Union Medical College Hospital & Chinese Academy of Medical Sciences & Peking Union Medical College, Beijing, China; 2 Department of Administrative Office, Haihe University, Tianjin, China; 3 Division of Liver Disease, The Fifth Hospital of Shijiazhuang, Hebei Medical University, Shijiazhuang, China; 4 Department of Cardiology, Beijing Hospital of the Ministry of Health, Beijing, China; 5 Department of Clinical Laboratory, Haihe University, Tianjin, China; 6 Department of Medicine, University of Hong Kong, Hong Kong, China

**Keywords:** SARS-CoV-2, Antibody, China

## Abstract

**Background:**

SARS-CoV-2 is a novel coronavirus first recognized in late December 2019 that causes coronavirus disease 19 (COVID-19). Due to the highly contagious nature of SARS-CoV-2, it has developed into a global pandemic in just a few months. Antibody testing is an effective method to supplement the diagnosis of COVID-19. However, multicentre studies are lacking to support the understanding of the seroprevalence and kinetics of SARS-CoV-2 antibodies in COVID-19 epidemic regions.

**Method:**

A multicentre cross-sectional study of suspected and confirmed patients from 4 epidemic cities in China and a cohort study of consecutive follow-up patients were conducted from 29/01/2020 to 12/03/2020. IgM and IgG antibodies elicited by SARS-CoV-2 were tested by a chemiluminescence assay. Clinical information, including basic demographic data, clinical classification, and time interval from onset to sampling, was collected from each centre.

**Results:**

A total of 571 patients were enrolled in the cross-sectional study, including 235 COVID-19 patients and 336 suspected patients, each with 91.9%:2.1% seroprevalence of SARS-CoV-2 IgG and 92.3%:5.4% seroprevalence of SARS-CoV-2 IgM. The seroprevalence of SARS-CoV-2 IgM and IgG in COVID-19 patients was over 70% less than 7 days after symptom onset. Thirty COVID-19 patients were enrolled in the cohort study and followed up for 20 days. The peak concentrations of IgM and IgG were reached on the 10th and 20th days, respectively, after symptom onset. The seroprevalence of COVID-19 IgG and IgM increased along with the clinical classification and treatment time delay.

**Conclusion:**

We demonstrated the kinetics of IgM and IgG SARS-CoV-2 antibodies in COVID-19 patients and the association between clinical classification and antibodies, which will contribute to the interpretation of IgM and IgG SARS-CoV-2 antibody tests and in predicting the outcomes of patients with COVID-19.

## Introduction

Coronavirus disease 2019 (COVID-19) has caused a great pandemic worldwide. As of October 12,020, more than 3 billion cases had been diagnosed with COVID-19, and the mortality rate was about 3.0% according to World Health Organization (WHO) reports. The challenges of this epidemics include treatment, avoiding viral transmission. The Chinese diagnostic and therapeutic guidelines of COVID-19 have been updated 7 times [1]. More detailed information on COVID-19 has been uncovered by clinical and basic research.

One of the dilemmas in the treatment of COVID-19 is the relatively high rate of false-negative results using nucleic acid tests as the diagnostic method. The reasons for this include several aspects, including a low viral concentration in the upper respiratory tract, unstandardized sample collection methods, various gene application performances, and a decrease in viral load one week after disease onset [2, 3].

Since an immune reaction is involved in COVID-19 progression, serological assays have been developed and put into practice in many countries [4]. Antibody tests have been confirmed as a good supplement for nucleic acid tests. They can be used as an immunity passport or proof of a previous infection, an asymptomatic infection or immunization. However, there are still many challenges and knowledge gaps in the clinical applications of antibodies in COVID-19 [5]. including the performances of various SARS-CoV-2 antibody products, the variable prevalences of antibodies in different regions, and the interpretation of positive results in various clinical stages. Previous studies on SARS-CoV-2 antibody tests in China were mostly conducted in a single centre or restricted to one province. Our study collected data from four epidemic cities during the outbreak stage of COVID-19 in China to better understand the significance of SARS-CoV-2 serological tests.

## Methods

### Patients and data collection

This study consists of a cross-sectional study and a cohort study. 235 confirmed COVID-19 patients and 336 suspected COVID-19 patients were identified in this multicentre study from Peking Union Medical College Hospital, Tianjin Haihe Hospital, the Fifth Hospital of Shijiazhuang, and Zhongnan Hospital of Wuhan University from 29/01/2020 to 12/03/2020. SARS-CoV-2 infection was confirmed by two repeated positive results from the local hospital using commercial RT-PCR kits for nasal and pharyngeal swab specimens. The suspected cases were defined as clinical manifestations, chest radiography imaging, and history of contacting COVID-19 confirmed patients. These suspected patients must have negative nucleic acid tests and the second test must be tested 1 day after the first negative nucleic acid test results. All patients were enrolled in the cross-sectional study. 30 patients in Tianjin were included in the cohort study to investigate the dynamic changes in IgM and IgG concentrations. All clinical data were retrieved from the Laboratory Information System and Hospital Information System from each centre. The ethics committee of Peking Union Medical College Hospital approved this study and waived informed consents for the usage of the remaining clinical samples (ZS-2303).

### Measurements

The remaining serum or plasma samples of included patients were collected after routine clinical tests. IgM and IgG antibodies elicited by SARS-CoV-2 in plasma and serum were tested immediately after sample collection by a chemiluminescence assay (CLIA, Lot Number: 20200127) developed by Beier Bioengineering Company (Beijing, China, http://www.beierbio.com/en/Default.aspx). The IgM antibody test was based on a μ-chain capture immunoassay, and the IgG antibody was detected by indirect immunoassays. Recombinant antigen-containing receptor-binding domain (RBD) of the SARS-CoV-2 spike protein and nucleocapsid (N) proteins were used to develop the IgM and IgG antibody assays. Horseradish peroxidase-conjugated mouse against human IgM/IgG antibody was used as the detection antibody. Both IgM and IgG tests were performed on an automatic chemiluminescence analyser (VI-200, Beier, Beijing, China) according to the manufacturer’s instructions. Cut-off values of IgM and IgG were 8 AU/ml. We clarify all methods used in our study comply with institutional, national, or international guidelines.

### Statistical analysis

Results were analysed using SPSS version 12.0 (SAS Institute, Cary, NC, USA). Continuous variables were expressed as mean with standard deviation for normally distributed data and the median with interquartile range (IQR) for skewed distribution data. Categorical variables are expressed as numbers (%). Kruskal-Wallis tests for continuous variables and chi-square tests for categorical variables were used for comparisons between groups. Patients were classified as light, regular, severe, and critical according to the Chinese Clinical Guidance for COVID-19 Pneumonia Diagnosis and Treatment (7th edition). They were stratified according to the time from disease onset to sampling into three groups: ≤7 days, 8–15 days, and > 15 days. Seroprevalence was defined as the proportion of patients with positive antibody results in the study. *P* < 0.05 was considered statistically significant. In the cohort study, the mean concentration of SARS-Cov-2 IgG and IgM were plotted with the time interval from disease onset to sampling.

## Results

### Baseline characteristics

A total of 571 patients were enrolled in this study, of which 144 patients were from Beijing, 147 patients were from Tianjin, 29 patients were from Wuhan, and 241 were from Shijiazhuang. The baseline characteristics of the 235 COVID-19 patients and 336 suspected patients are summarised in Table 1. The average interval from symptom onset to COVID-19 testing in patients in Wuhan was 20.9 days, which was longer than that at other study sites, while the time interval in Beijing was the shortest at 3.3 days. Suspected patients in Beijing and Tianjin were generally older than the confirmed COVID-19 patients. However, suspected patients in all hospitals was younger than that of the confirmed COVID-19 patients for patients from Shijiazhuang. There were relatively more male patients in this study. 216 (91.9%) and 217 (92.3%) were IgG positive and IgM positive in COVID-19 patients, respectively. In 336 suspected patients, 7 (2.1%) tested IgG positive, and 18 (5.4%) tested IgM positive.

**Table 1:** Demographic characteristics of the patients enrolled in the cross-sectional study

		Beijing	Tianjin	Wuhan	Shijiazhuang	Sum	*P* value
COVID-19 confirmed patients	Cases	12	127	29	67	235	–
	average interval from onset to sampling (days)	3.3	10.4	20.9	13.5	–	–
	Age	40.0 (34.0,63.0)	49.0 (36.0,61.0)	61.0 (46.5,69.0)	44.0 (34.0,65.0)	49.0 (35.0,64.0)	0.048
	Male	5 (41.7%)	64 (50.4%)	16 (55.2%)	45 (67.2%)	130 (55.3%)	0.114
	seroprevalence of IgG	75.0%	95.3%	75.9%	95.5%	91.9%	< 0.001
	seroprevalence of IgM	75.0%	95.3%	75.9%	97.0%	92.3%	< 0.001
Suspected patients	Cases	132	20	–	184	336	–
	Age	45.5 (29.0,63.0)	63.5 (50.8,70.8)	–	33.0 (26.0,44.0)	37.0 (28.8,56.0)	< 0.001
	Male	61 (46.2%)	12 (60.0%)	–	96 (59.8%)	183 (54.5%)	0.051
	seroprevalence of IgG	3.8%	0.0%	–	1.1%	2.1%	< 0.001
	seroprevalence of IgM	13.1%	0.0%	–	0.5%	5.4%	< 0.001

*P* < 0.05 was considered a significant difference between groups

### Antibodies concentration

#### Antibody concentrations stratified by clinical classification

Forty-one COVID-19 patients with clinical classification information from Beijing and Wuhan were analysed. Characteristics of these patients are shown in Table 2. Although there was only one patient with very mild symptoms, there was an increase in median age, concentration, and positive rate of IgG and IgM with aggravation of the illness.

**Table 2:** Antibody concentrations stratified by the clinical classification

	Clinical Classification			Sum	*P* value
	Mild	Regular	Severe		
N	1	34	6	41	–
Age	25	52.5 (39.8, 64.8)	67.0 (55.5,71.5)	60.0 (39.5,67.5)	0.091
Male	0.0%	50.0%	33.3%	51.2%	0.366
Concentration of COVID-19 IgG (AU/ml)	9.1	22.2 (3.9, 89.4)	157.9 (28.8190.7)	30.6 (8.4, 129.4)	0.056
Seroprevalence of COVID-19 IgG	100.0%	70.6%	100.0%	65.9%	0.256
Concentration of COVID-19 IgM (AU/ml)	8.17	27.9 (4.4, 145.6)	29.7 (8.6, 188.6)	25.5 (5.2152.7)	0.677
Seroprevalence of COVID-19 IgM	100.0%	73.5%	83.3%	75.6%	0.742

Seroprevalence was defined as the proportion of patients with positive antibody results in the study

*P* < 0.05 was considered a significant difference between groups

### Comparison of SARS-Cov-2 IgG and IgM level in COVID-19 patients between regions and symptom onset intervals

**Fig. 1:**
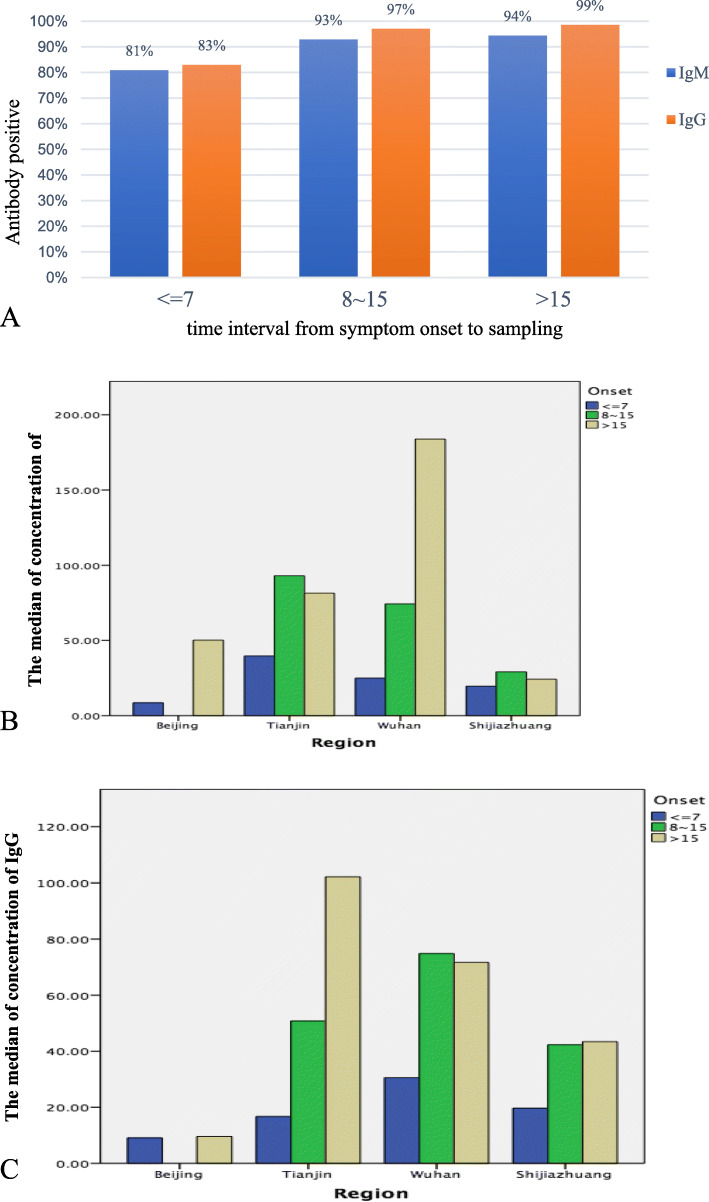
The seroprevalence and concentration comparison of COVID-19 IgG and IgM in diagnosed patients stratified by region and symptom onset interval. **a** The seroprevalence of COVID-19 IgG and IgM stratified by symptom onset interval. **b** The seroprevalence of COVID-19 IgM stratified by symptom onset interval and region. **c** The seroprevalence of COVID-19 IgG stratified by symptom onset interval and region

We collected the time interval from symptom onset to sampling in all COVID-19 patients in the cross-sectional study. The SARS-Cov-2 IgG- and IgM-positive rates stratified by the time interval are shown in Fig. 1a. The positive rate gradually increased with the increase in testing time interval from symptom onset. The positive rate of SARS-Cov-2 IgG was close to 100% at > 15 days. Figure 1b and c shows the median concentrations of SARS-Cov-2 IgG and IgM in various regions. The concentrations of SARS-Cov-2 IgG and IgM rose after 7 days of onset in all hospitals. The concentrations of SARS-Cov-2 IgM in patients from Wuhan and IgG in patients from Tianjin ascended distinctively step by step along with the time intervals.

#### The dynamic characteristics of SARS-Cov-2 IgM and IgG in the cohort study

**Fig. 2:**
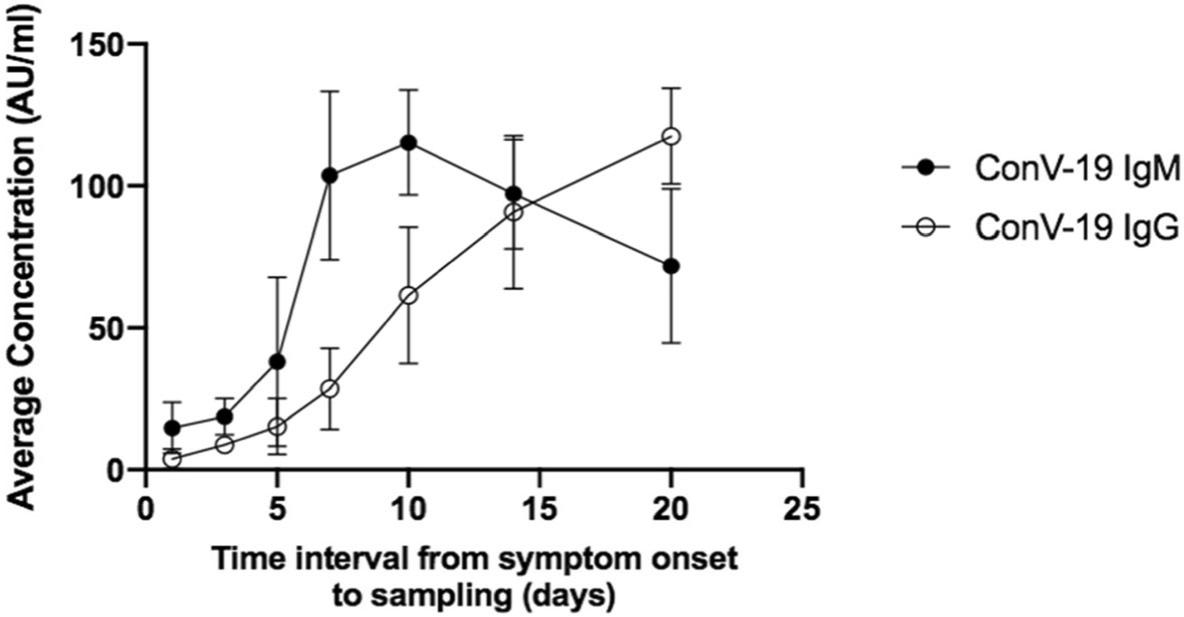
The kinetics of antibodies against SARS-CoV-2 in continuously monitored patients with COVID-19 disease

The dynamic changes in SARS-Cov-2 IgM and IgG concentrations over time were investigated in 30 COVID-19 patients from Tianjin. The dynamic change of SARS-Cov-2 IgM and IgG are shown in Fig. 2. The concentration of SARS-Cov-2 IgM was higher than that of IgG before the 15th day after symptom onset. The concentration of SARS-Cov-2 IgG was higher than that of IgM after 15th day since symptom onset. The SARS-Cov-2 IgM concentration reached a peak on the 10th day after symptom onset and then decreased slowly. The concentration of SARS-Cov-2 IgG on the 20th day continued to increase.

## Discussion

This is the first multicentre study of antibodies against SARS-COV-2 from four outbreak areas in China. We found a good clinical coincidence rate of the antibody tests, an association between the clinical classification and the concentration of antibodies, and the kinetics of the antibodies, which could improve our understanding in the immune response after patients are infected with SARS-CoV-2. In this study, we investigated the seroprevalence of SARS-Cov-2 IgG and IgM as well as the kinetics of the antibody response in COVID-19 from four epidemic regions of China in early 2020 [6]. In the cross-sectional study, 91.9 and 92.3% of patients with confirmed SARS-CoV-2 infection tested IgG and IgM positive, respectively. 2.1% of 336 suspected patients tested IgG positive, and 5.4% tested IgM positive. In the cohort study, the peaks of IgM and IgG were reached on the 10th and 20th days, respectively, after symptom onset. The seroprevalence of SARS-CoV-2 IgG and IgM increased along with the clinical classification and treatment time delay.

There has been an urgent need for novel in-vitro diagnostic products. The sensitivity and specificity of these products ranged 88–100% and 75–100%, respectively [7–9]. Sensitive and stable CLIA was used as a measurement method in our study. Figure 1a shows that the positive antibody rate increased along with the symptom onset intervals. The seroprevalence of SARS-CoV-2 IgM and IgG on ≤7 days since onset of symptoms were 81 and 83%, respectively. The seroprevalence of both antibodies raised to 95% at 2 weeks since symptom onset [10].

Approximately 8% of COVID-19 patients tested negative for IgM or IgG. We speculated three explanations which might contribute to the negative results for confirmed COVID-19 patients. First, the heterogeneity of testing times from the onset of the disease are an important factor [11, 12]. Previous study showed that antibodies elicited by SARS-CoV-2 develop three days after symptom onset or one week after infection with SARS-CoV-2 [13]. In our study, the average time interval from onset to sampling was 3 days in Beijing, followed by Tianjin, Shijiazhuang, and Wuhan in an increasing order of average time interval (Table 1). Therefore, the seroprevalences of IgM and IgG antibodies in Shijiazhuang and Tianjin were higher than those in Beijing and Wuhan. Therefore, antibody testing during the window phase of COVID-19 progression could lead to false-negative results [14, 15]. Secondly, individual differences in the immune response are also a contributing factor. Some COVID-19 patients were negative for SARS-CoV-2 IgM and IgG from onset to recovery [16], which indicates that innate immunity could clear the virus without adaptive immunity and these patients might not produce detectable antibodies against SARS-CoV-2 [15, 16]. Additionally, the sample size of COVID-19 patients was limited in Beijing and Wuhan, which might lead to underestimation of the seroprevalence [17].

On the other hand, 2.1 and 5.4% of patients were found SARS-CoV-2 IgG and IgM positive in suspected patients. Many causes of false-positive results have been reported, including autoimmune disease, cancer, drug usage, and other infections [18, 19]. Therefore, antibody tests are recommended in combination with nucleic acid tests for the diagnosis and treatment of COVID-19.

The association of SARS-CoV-2 antibody concentration, positive rate, and clinical stage was explored in our study. Although there was no statistical significance due to limited number of patients included in this study, SARS-CoV-2 IgM and IgG antibody concentrations and positive rates in severe cases were distinctively higher than those in milder cases (Table 2). Our study further confirmed the findings from the previous study. Long et al. and Qu et al. indicated that critical COVID-19 patients had higher IgM and IgG antibody responses than non-critical patients [20, 21] due to a high level of viral load or inflammatory storm in severe or critical cases [22]. In addition, we found that the mean ages of COVID-19 patients with severe cases were older than those with milder cases, although the difference was not statistically significant. Many studies have reported that patients who died were generally older than survivors in critical cases of COVID-19 [23–25], especially among patients with comorbid diseases, including hypertension, coronary artery disease, and diabetes [25, 26]. Age is one of the risk factors for susceptibility and poor prognosis of COVID-19 [27].

The kinetics of SARS-CoV-2 IgM and IgG in COVID-19 patients are shown in Fig. 2. The IgM antibody concentration reached a peak 10 days earlier than the IgG antibody concentration. The SARS-CoV-2 IgG antibodies maintained an upward trend after 20 days. Andrea et al. reported that IgM antibody levels peaked at 10–12 days and significantly declined after 18 days [28] which was similar to our study. IgG against COVID-19 has been reported to persist over seven weeks [11]. Some studies showed that COVID-19 patients with high IgG titres might produce neutralizing antibody activity, clearing the virus [29, 30]. Wang et al. reported a moderate correlation between anti–SARS-CoV-2 spike protein IgG levels and neutralization titres in COVID-19 patient plasma [5]. In contrast, some studies observed higher levels of anti-RBD IgG antibodies from COVID-19 patients that did not contribute to neutralization. They suggest that anti-RBD IgM and IgA also contribute to neutralization [31, 32]. Since the virus-neutralizing antibody titre was determined by the virus infection inhibition rate, the content of neutralizing antibodies in the serum was found to be complex and is being recognized gradually [33]. The detection antibodies in commercial reagents usually target spike and/or nucleocapsid proteins and may not distinguish among different immunogenic regions of the spike protein of SARS-CoV-2 [34]. Therefore, predicting whether serum with positive antibodies is protective or therapeutic should be approached with caution.

Nevertheless, there were some limitations in our study. First, the limited sample size and clinical information in some regions restricted more analysis to perform. Additionally, individuals with asymptomatic SARS-CoV-2 infection were not included in our studies, so information from those patients was lacking.

In conclusion, we demonstrated the seroprevalence of SARS-CoV-2 IgM and IgG antibody and antibody titre alterations in COVID-19 patients, which could help in better interpreting the antibody testing results during COVID-19 progression.
